# Protocol for a phase III RCT and economic analysis of two exercise delivery methods in men with PC on ADT

**DOI:** 10.1186/s12885-018-4937-x

**Published:** 2018-10-23

**Authors:** Shabbir M. H. Alibhai, Paul Ritvo, Daniel Santa Mina, Catherine Sabiston, Murray Krahn, George Tomlinson, Andrew Matthew, Himu Lukka, Padraig Warde, Sara Durbano, Meagan O’Neill, S. Nicole Culos-Reed

**Affiliations:** 1 University Health Network, Toronto, ON M5G 2C4 Canada; 2 University of Toronto, Toronto, ON M5S 2J7 Canada; 3 Cancer Care Ontario, Toronto, ON M5G 2L3 Canada; 4 The Juravinski Cancer Centre, Hamilton, ON L8V 5C2 Canada; 5 University of Calgary, Calgary, AB T2N 1N4 Canada; 6 Toronto General Hospital, 200 Elizabeth St Room EN14-214, Toronto, ON M5G 2C4 Canada

**Keywords:** Prostate cancer, Androgen deprivation therapy, Exercise, Randomized controlled trial, Quality of life, Fatigue, Physical fitness, Cost-effectiveness, Patient adherence

## Abstract

**Background:**

Androgen deprivation therapy (ADT) is commonly used to treat prostate cancer. However, side effects of ADT often lead to reduced quality of life and physical function. Existing evidence demonstrates that exercise can ameliorate multiple treatment-related side effects for men on ADT, yet adherence rates are often low. The method of exercise delivery (e.g., supervised group in-centre vs. individual home-based) may be important from clinical and economic perspectives; however, few studies have compared different delivery models. Additionally, long-term exercise adherence and an understanding of predictors of adherence are critical to achieving sustained benefits, but such data are lacking. The primary aim of this multi-centre phase III non-inferiority randomized controlled trial is to determine whether a home-based delivery model is non-inferior to a group-based delivery model in terms of benefits in fatigue and fitness in this population. Two other key aims include examining cost-effectiveness and long-term adherence.

**Methods:**

Men diagnosed with prostate cancer of any stage, starting or continuing on ADT for at least 6 months, fluent in English, and living close to a study centre are eligible. Participants complete five assessments over 12 months (baseline and every 3 months during the 6-month intervention and 6-month follow-up phases), including a fitness assessment and self-report questionnaires. Biological outcomes are collected at baseline, 6, and 12 months. A total of 200 participants will be randomized in a 1:1 fashion to supervised group training or home-based training supported by smartphones, health coaches, and Fitbit technology. Participants are asked to complete 4 to 5 exercise sessions per week, incorporating aerobic, resistance and flexibility training. Outcomes include fatigue, quality of life, fitness measures, body composition, biological outcomes, and program adherence. Cost information will be obtained using patient diary-based self-report and utilities via the EQ-5D.

**Discussion:**

To disseminate publicly funded exercise programs widely, clinical efficacy and cost-effectiveness have to be demonstrated. The goals of this trial are to provide these data along with an increased understanding of adherence to exercise among men with prostate cancer receiving ADT.

**Trial registration:**

The trial has been registered at clinicaltrials.gov (Registration # NCT02834416). Registration date was June 2, 2016.

**Electronic supplementary material:**

The online version of this article (10.1186/s12885-018-4937-x) contains supplementary material, which is available to authorized users.

## Background

Prostate cancer (PC) is the most common cancer in men [1, 2], affecting an estimated 21,600 Canadian men in 2016. With increased screening and better treatment of early disease, 10-year disease-specific survival has reached almost 95% [2]. This means that more men are living longer with PC, with many progressing to advanced disease over time. Androgen deprivation therapy (ADT), an effective treatment, blocks the production of testosterone, leading to apoptosis and decreased proliferation of PC cells. It has been used in advanced PC for 7 decades [3]. At present, almost 50% of men with PC can expect to receive ADT at some point after diagnosis [4–6]. Although ADT improves disease control and prolongs survival (often by several years) [7–9], it is associated with numerous adverse effects, predominantly due to depriving organs and tissues of testosterone. These adverse effects include worsening quality of life (QOL) and fatigue [10–13], significant declines in muscle mass and physical strength, loss of bone mineral density (BMD), and various metabolic side effects (e.g. increased blood glucose and cholesterol).

The effects of ADT on health-related QOL (hereinafter referred to as QOL), are diverse and persistent, including worse global QOL, worse physical function, more fatigue, poorer sexual function, hot flushes, and breast tenderness [14–24]. Some studies have reported worse social function [15] and mental well-being [15, 18]. These effects are typically seen within 3 to 12 months of ADT initiation [12] and persist or slowly worsen over the next 3 years with ongoing ADT use [25]. Such effects are profoundly disruptive to patients, spouses or partners, and families [10, 11, 26–28].

Multiple studies have reported reductions in overall muscle mass, upper and lower extremity strength, grip strength, and daily functioning in men on ADT [12, 24, 29–36]. Two studies suggested an increased risk of falls in men on ADT, most of whom are elders [37, 38]. Numerous studies have also shown reductions in BMD within 6–12 months of ADT initiation, with further declines with ongoing ADT use for at least 10 years [39–46]. Loss of BMD has been associated with a 40–60% increased risk of fracture [13, 47–50]. Finally, ADT has also been associated with increased fat mass, insulin resistance, increased blood glucose, and unfavourable alterations in lipids [34, 35, 51–55]. These metabolic changes are associated with an increased risk of diabetes [13, 56–58] and cardiovascular outcomes [13, 56, 57, 59–63], although the latter remains controversial [64, 65].

Exercise-based interventions have been shown in at least 14 clinical trials to ameliorate most of these adverse effects [30, 66–77]. As summarized in several recent systematic reviews [78–82], the benefits of exercise have been shown in interventions of 8 to 24 weeks’ duration, including improved general or overall QOL [30, 70, 74], prostate-specific QOL [30, 74], and fatigue [30, 66, 69, 70, 73, 74]. Exercise has also been associated with improved muscular endurance (upper body = 22–115%, lower body = 23–167%), increased maximal strength (upper body = 40%; lower body = 96%), increased quadriceps muscle thickness by 16%, preserved or improved total body lean mass, and prevention of fat gain [30, 68, 70, 73]. No other intervention has been shown to improve as many side effects of ADT.

Three small studies found preliminary evidence of benefits of exercise in insulin parameters, glucose, lipids, or inflammatory markers, but effects on BMD remain unclear [73, 74, 83]. These effects need to be confirmed in larger studies, given the potential benefits of preventing diabetes or reducing cardiac risk factors in older men on ADT, the majority of whom will die of heart disease rather than PC [84–91]. Importantly, there is a robust literature in the general population showing that exercise improves glucose [92–94], lipids [95], and BMD [96–98]. And regular exercise after a diagnosis of PC may be associated with improved survival [99].

Although multiple studies have demonstrated many benefits of exercise for men with PC on ADT, the strongest and most consistent benefits have been shown with 1:1 supervised (i.e. personal training), in-centre interventions [100]. As summarized in recent reviews [81, 82, 101], four trials have featured 1:1 supervised exercise interventions, all of which reported positive effects on QOL or fitness outcomes. Two studies (only one was randomized) were group-supervised interventions, and both showed benefits in QOL and/or fitness outcomes. In contrast, of the four home-based studies (two randomized controlled trials (RCTs)), only two were positive, and the magnitude and breadth of benefits were smaller than with supervised exercise interventions. Of note, these three exercise delivery models have not been directly compared to one another. Prior trials have used different outcome measures, assessment time points, and training routines, making indirect comparisons difficult.

Despite the accumulating evidence of benefits, few men with PC, including those on ADT, exercise regularly [99, 102–105]. For exercise interventions to be most useful to men on ADT, long-term exercise adherence and benefits must be achieved. Long-term exercise adherence has been defined as engaging in regular exercise for at least 6 months post-intervention [106]. In the general population, about 50% of individuals stop exercising after 6 months. Experience with cancer survivors suggests that they fare no better [107]. Cessation of exercise leads to loss of benefits in virtually every setting [108]. Unfortunately, most previous research in men on ADT has been limited to reporting exercise benefits and adherence only to the end of the supervised exercise intervention [79, 80]. In two studies examining participants 4–6 months post intervention, weekly exercise was reduced and QOL benefits were diminished over time [69, 74].

Relatively little is known about what factors are associated with adherence in this population. Only one study has formally examined adherence in an exercise intervention in men on ADT, finding that age, exercise stage of change, and intention were independent predictors of adherence [109]. However, only supervised in-centre exercise was examined and adherence was assessed only during the intervention phase. In summary, although adherence to exercise is crucial, little is known about adherence with different exercise delivery models and factors predicting adherence after the formal intervention phase in men on ADT. It is particularly important to identify factors that support long-term adherence, since men on ADT often continue this treatment for at least 2 to 3 years (and often indefinitely, depending on the indication).

Two other important barriers to exercise uptake relate to resources. First, despite its clinical value, no publicly funded exercise program exists in Canada for anyone with a cancer diagnosis, including men on ADT. This is likely due, in part, to the absence of any data on cost-effectiveness of exercise interventions in patients with PC. In contrast, cardiac rehabilitation is widely available and publicly funded because it has been shown to be both efficacious and cost-effective [92, 110]. Given increasing fiscal pressures, all levels of government are carefully evaluating both efficacy and cost-effectiveness of potentially insurable services.

Second, a related resource issue is alternate exercise delivery models. As outlined above, three main exercise delivery models have been studied in men on ADT – supervised 1:1, group-supervised, and home-based programs; however, our group has conducted the only clinical trial directly comparing 2 exercise delivery models (supervised 1:1 and group supervised) in men on ADT [77]. This trial was a pilot study that demonstrated the feasibility of enrolling patients into a RCT of different exercise modalities along with high intervention adherence and study retention [77]. However, multiple comparative trials have been conducted in other settings [111, 112]. For example, a systematic review of 12 RCTs of almost 2000 patients in cardiac rehabilitation has shown similar benefits of home-based versus in-centre rehabilitation on exercise capacity, cardiac events, modifiable risk factors (lipids, blood pressure, etc.), and mortality [112]. Of note, adherence was greater among home-based compared to in-centre participants in those studies. Clearly, both models require significantly fewer resources for population-level delivery than the gold-standard of 1:1 supervised, facility-based training. In addition, these models of exercise delivery may have added benefits such as improved adherence or social interaction, yet these modalities have never been effectively and directly compared for clinical efficacy or cost-effectiveness in men on ADT.

Given the gaps in the published evidence, we recently conducted a phase II trial to assess the feasibility and efficacy of these 3 delivery models in a 6-month intervention [113] (manuscript submitted). The preliminary results suggested that group-supervised exercise and home-based exercise were generally non-inferior to 1:1 supervised exercise for both QOL and fitness outcomes. Specifically, group-supervised exercise was shown to have less than a 20% probability of being inferior to 1:1 exercise for 2 of 3 QOL and 3 of 3 fitness outcomes. The home-based arm had less than a 25% probability of being inferior to the 1:1 arm for 1 of 3 QOL and 3 of 3 fitness outcomes. Moreover, the home-based arm was associated with the highest adherence based on accelerometry. Altogether, this provided a foundation to refine our phase II protocol and conduct the present phase III trial, in which we decided to directly compare 2 exercise delivery methods that are least resource intensive. Although efficacious, the 1:1 supervised intervention arm appeared to provide minimal benefit over a group-supervised program and is cost-prohibitive within a publicly funded health care system.

The primary aims of this phase III non-inferiority RCT are:

i. To determine, in men with PC on ADT, whether a home-based supported exercise program is non-inferior to a group supervised in-centre (facility-based) exercise program on the primary outcomes of fatigue and functional endurance;
ii. To examine adherence to exercise and predictors of adherence in each exercise group during the 6-month intervention and for 6 months after program completion;
iii. To conduct an economic analysis comparing both exercise interventions and usual care.

## Methods

### Study design

**Fig. 1:**
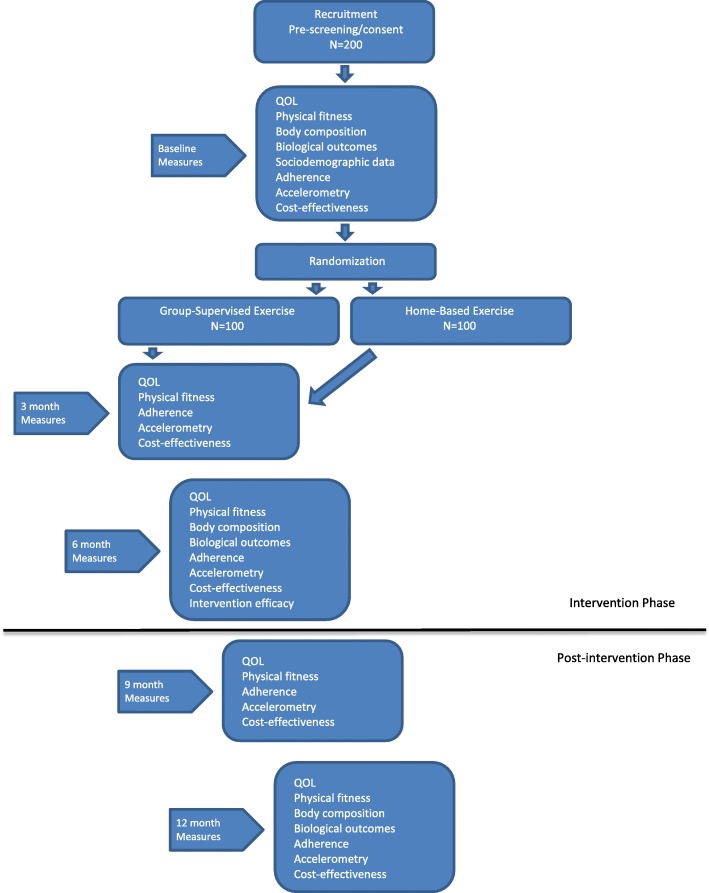
Study flow diagram

This trial is a multi-centre, 2-arm non-inferiority RCT with blinded, validated, and clinically relevant outcome measures (Fig. 1). It will take place at two of Canada’s largest, university-affiliated cancer centres with large genitourinary site groups and significant RCT and exercise expertise: the Princess Margaret Cancer Centre (PM) in Toronto and the Tom Baker Cancer Centre in Calgary (TBCC). Participants will also be recruited from 2 community-based hospitals (in the Greater Toronto Area), Southlake Regional Health Centre and Scarborough and Rouge Hospital – Centenary site. Ethics approval has been obtained at all institutions. All study participants will provide written informed consent prior to study enrollment. Participants can voluntarily withdraw from the study at any time. Participants will be withdrawn from the study at the time of symptomatic disease progression. Any protocol amendments will be approved by the research ethics boards and communicated to investigators as well as to study participants as directed by the research ethics board. The trial is registered at clinicaltrials.gov (registration number: NCT02834416) and the SPIRIT figure is shown in Additional file 1.

### Study participants

The trial will include men with histologically confirmed PC who are fluent in English, able to provide consent, live close to a study centre and are either starting or continuing on ADT for at least 6 months or those in an androgen-deprived (or castrate) state for the duration of the intervention (total testosterone < 1.7 nmol/L). Men are ineligible if they are already meeting guidelines for moderate to vigorous physical activity (MVPA) or have conditions that would interfere with ability to participate. Details are listed in Additional file 2: Table S1.

### Recruitment

Each study site will feature a dedicated research coordinator (RC) who is a Certified Exercise Physiologist (CEP) or Registered Kinesiologist (RKin). Attending PC specialists (urologists, radiation oncologists, and medical oncologists) in PC clinics will identify potentially eligible patients and notify the RC. The RC will approach potential patients and a study information package will be provided. For patients interested in participating, written informed consent will be obtained and a baseline assessment will be scheduled. Based on patient volumes per centre and prior trial experience, we estimate recruiting 200 patients in approximately 24 months.

### Assessments

Participants will complete 5 assessments over the course of the trial (baseline, 3, 6 (end of intervention), 9, and 12 months). The 9- and 12-month assessments are completed following the end of the intervention and will provide information following the active intervention period. Outcome assessments are completed by a trained outcome assessor. Although neither the patient nor the exercise leader can be blinded to treatment allocation, outcome assessors will be blinded. At each assessment time point, participants will receive compensation to subsidize parking costs and to acknowledge their ongoing commitment to the trial.

### Baseline assessment

The baseline assessment consists of obtaining socio-demographic and clinical data as well as assessing fitness using measures of resting heart rate (HR), blood pressure (BP), oxygen saturation, body composition, upper and lower body strength testing, and a measure of functional endurance. Participants will also complete a series of questionnaires (to assess QOL, fatigue, and exercise adherence, including prior exercise program participation [114]) and will be scheduled to complete both a BMD test (if one has not been completed within the past year as per standard of care) and blood work. All measures are detailed below and summarized in Table 1. First aid and emergency equipment and supplies will be on hand for all testing and training sessions.

**Table 1:** Summary of Study Measures at Specified Time Points

Domain/Measure	Time required	T_0_: (Baseline)	T_1_: (3 mo.)	T_2_:6 mo. (End Int.)	T_3_: 9 mo. (3 mo. f/u)	T_4_:12 mo. (6 mo. f/u)
Quality of life						
FACT-P	4–5 min	●	●	●	●	●
FACT-G	8–10 min	●	●	●	●	●
FACT-F (co-primary)	5 min	●	●	●	●	●
Physical Fitness						
6MWT (co-primary)	6 min	●	●	●	●	●
Sit-to-Stand Test	1 min	●	●	●	●	●
Grip Strength	1 min	●	●	●	●	●
Biological Outcomes^a^	< 5 min	●		●		●
Blood glucose						
Cholesterol profile						
PSA (safety)						
Testosterone^c^						
Haemoglobin (covariate)						
HbA1c (covariate)						
Body Composition						
Bone mineral density	30 min^b^	●				●
Body composition^d^	5 min	●		●		●
Adherence						
Accelerometer	–	●	●	●	●	●
GLTEQ	< 5 min	●	●	●	●	●
Sessional attendance^e^	–					
Adherence Predictor Variables						
HCCQ	5 min	●				
BREQ2	5 min	●		●		●
PNSF	5 min	●		●		●
PAB	5 min	●		●		●
Sedentary Behaviours	5 min	●		●		●
Cost-Effectiveness						
Health questionnaire	5 min	●	●	●	●	●
EQ-5D	5 min	●	●	●	●	●
Study Completion	5 min					●

^a^ All biological measures are considered to be standard of care at baseline and 12 months. PSA is considered to be standard of care at all 3 time points. Blood glucose, cholesterol profile, and haemoglobin tests at 6 months are NOT standard of care

^b^ Can be done on separate day to reduce participant burden

^c^ Will only be measured in participants who are not on continuous ADT for the duration of the intervention

^d^ Includes BIA, waist circumference, and waist circumference: height ratio

^e^ Only for those in supervised groups (done weekly)

*Abbreviations***:** *6MWT* 6 min walk test, *BREQ2* Behavioral Regulations in Exercise Questionnaire – 2, *EQ-5D* EuroQol 5 dimensions of health scale, *FACT-G* Functional Assessment of Cancer Therapy General, *FACT-F* Fatigue subscale, *FACT-P* Prostate subscale, *GLTEQ* Godin Leisure Time Exercise Questionnaire, *HbA1c* glycated hemoglobin, *HCCQ* Health Care Climate Questionnaire, *Int* Intervention, *PAB* Planning, Attitudes, & Behaviour questionnaire, *PSA* prostate-specific antigen, *PNSF* Psychological Need Support and Frustration Scale – Relatedness Items

### Randomization

Patients will be allocated in a 1:1 ratio to treatment groups through a web-based central randomization scheme, with stratification on centre and prior duration of ADT (**<** 3 months vs. 3+ months). We are stratifying on duration of prior ADT as this has been shown to impact on subsequent declines in physical function and QOL as well as response to exercise [12, 115]. Randomization will occur following the baseline assessment. Once randomized, group-supervised participants will be scheduled to attend their first exercise session and home-based participants will be scheduled to attend an orientation session conducted by the RC and health coach. All participants will receive a study manual and basic exercise equipment (stability ball, mat, and resistance bands) to support program adherence.

### Follow-up assessments

All follow-up assessments will include documentation of any change in clinical status as well as completion of the fitness measures described above and self-report questionnaires. BMD testing will occur only at baseline and 12 months and body composition measures and blood work will be collected at baseline, 6, and 12 months (Table 1).

### Intervention

The 6-month intervention consists of two delivery arms, a group-supervised arm and a home-based arm. Both programs are designed with evidence from previous research by our team [30, 69, 70, 72, 76, 77, 116] and will be individualized using baseline fitness results. Prior to starting the intervention, the CEP/RKin will meet with participants to provide instruction regarding the exercise program. All participants will be asked to complete 4–5 days per week of mixed modality exercise incorporating flexibility (stretching for 5–10 min at the end of each session), aerobic exercise (approximately 30 min targeting 60–70% heart rate reserve (HR_reserve_) assessed by heart rate monitor), and resistance training (using body weight, resistance bands and/or free weights, a stability ball, and/or an exercise mat with prescribed exercises that target the major muscle groups), encouraging participants to work toward a target of 150 min per week as per established guidelines [117]. The absolute workload (60–70% HR_reserve_), and time is standard across all interventions. Program adaptation and progression are described below and summarized in Table 2.

**Table 2:** Exercise Program Details

	Group-Supervised Exercise	Home-Based Exercise
Frequency of exercise	4–5 days per week (see also Delivery Location below)	
Intensity	AET: RPE of 11–14/20; HR of 60–70% of HRR RET: RPE of 11–14/20; 60–75% of 1RM × 8–12 repetitions × 2–3 sets; 5 exercises/session alternating each day (10 different RET exercises in total)^a^	
Session Duration	Identical	
Delivery Location of exercise		
*Facility-based*	3 x/week	Not applicable
*Home-based*	1–2 x/week	4–5 x/week
Trainer Presence	Group Exercise Leader	Unsupervised; remote support from health coach

*1RM* one-repetition maximum (maximum amount of weight that can be lifted), *AET* aerobic exercise training, *HRR* heart rate reserve, *RPE* Rating of perceived exertion, *RET* resistance exercise training

^a^All resistance exercises are conducted using body weight or resistance bands. A stability ball and yoga mat will also be available for use. RET exercises will target different major muscle groups delivered in two alternating programs. Day 1: Chest, upper back, shoulders, and arms. Day 2: Legs, gluteals, mid back, and core

Each program also includes an education component of 12 topics that focus on common concerns facing new exercisers (Table 3). Education sessions will occur during in-centre group classes for group-based participants and over the phone or during monthly in-person sessions for participants in the home-based arm of the intervention.

**Table 3:** Education Topics

Education Topics	Key Points
1) Introduction to Exercise	● Benefits of Physical Activity ● Program targets 3 areas of PA (aerobic, resistance, and flexibility) ● PA is safe, feasible and has shown to provide benefits
2) Goal Setting	● Goal setting will assist with your dedication and motivation to complete the exercises ● SMART Goals - Specific, Measurable, Attainable, Realistic, Timely ● Use the goal worksheet in the manual ● Make long term and short term goals
3) Behavior Change	● The plan you set out may not be followed 100% ● Anticipate obstacles that may come as you are changing a behaviour and develop strategies for dealing with it before it arises. ● Monitor your progress, Reward yourself, Visualize your success
4) Planning for Barriers	● Biggest perceived obstacles ○ Lack of time, self-discipline, partner and ability ● Plan ahead for periods of inactivity
5) Social Support	● You are more likely to be successful if your family, friends and even co-workers are supportive of you ● Social support can occur in many forms – encouragement, completing activities with you, etc.
6) Monitoring Behavior	● Mix up your activities to stay motivated ● Try something new, or something you have done prev. ● It is very easy to enter an exercise rut
7) Maintaining Motivation	● Greatest source of motivation: Fun/enjoyment/stimulation, feeling of accomplishment, pleasure of learning and benefits (i.e. improved sleeping) ● Pursue something that you enjoy, that is convenient to your schedule. ● Take opportunities to be active
8) Personal Control	● Believing that you are in control of your own life give you reinforced motivation and further commitment to make changes
9) Self- Discipline, Reward & Attitude	● Self-discipline can result in increased productivity, improved self-esteem and confidence ● Rewards – use workbook in manual ● Attitudes toward change can determine whether you will be successful
10) Adapting your Program	● Adapting your program – FITT principle
11) Health and the Media	● Be mindful of the ‘Get fit quick’ media marketing – Healthy eating and regular PA will help maintain a long-term healthy lifestyle
12) Lifelong Active Living	● Use some of the tips and tricks in the manual to assist with continuing your active life. ● Change things up, work towards small goals, work with a friend, etc.

Group-based exercise program delivery will be standardized across sites using several approaches. Training staff (CEPs/RKins) will be required to attend an introductory training session covering participant testing and training parameters, safety precautions, and emergency procedures. An exercise manual will be developed for training staff outlining specific exercises and appropriate progression of intensity. Monthly conference calls will be held with CEPs/RKins and RCs across all sites to ensure standardization and solve problems. Finally, trainers will record weekly exercise plans and logs for each participant or group. These will be audited by the site PIs and randomly audited by the study PI and central RC monthly for the first 6 months and then every 3 months if compliance is > 90% with the training manual.

#### Monitoring intensity and progression

The 10-point Rating of Perceived Exertion (RPE) scale will be used to monitor exercise intensity during each exercise session [118]. Participants will be instructed to exercise at a level of 3–6 on the 10-point RPE scale (12–16 on the 15-point scale). This corresponds to the target HR range detailed above and is a suitable alternative to regular HR monitoring [119]. In addition, HR monitors (Polar, NY, USA) will be used at 3-week intervals throughout the intervention to ensure that the intensity of aerobic exercise being performed aligns with the absolute workload prescribed and to monitor individual exercise progression. If a participant’s HR is outside of his target HR range, exercise intensity will be modified to ensure training within the target HR zone. Participants who need to increase their aerobic exercise workload will first increase exercise duration (e.g. walking minutes), followed by the intensity of exercise (e.g., walking speed). If a participant is able to perform ≥12 repetitions and 3 sets of any given resistance training exercise, the resistance level used for that exercise will be increased (e.g., from a medium to a heavy band). Participants in the group-supervised arm of the intervention will wear a HR monitor during group exercise classes as indicated. Participants in the home-based arm of the intervention will be given a HR monitor to use for the duration of the intervention and will be taught how to use and read their HR response, wearing the monitor as instructed by their health coach.

#### Group-supervised program

Participants in the group-supervised program will take part in classes led by a CEP/RKin 3 times per week for a period of 6 months. At each site, groups will commence as soon as 2 men have been randomized to a group and will grow to include 4–8 participants to ensure adequate supervision and progression. Participants will be provided with a set of resistance bands to support additional independent exercise.

#### Home-based program

Participants in this arm will receive equipment (exercise bands, ball, mat, heart rate monitor) free of charge to support home exercise. The frequency, intensity, time, and type of exercise are the same as in the group-supervised program although specific exercises in the aerobic component may be modified to incorporate participant preference and interest.

Since the strongest evidence in terms of exercise delivery appears to support supervised programs [78, 100], we have incorporated enhancements to prior home-based programs [66, 69, 72] based on emerging data in the field of behavioural interventions [120–122] as well as data from our phase II trial [101]. In particular, we will employ cutting-edge smartphone technology and the use of Fitbits as well as remote ‘health coaches’ to provide support to patients during the intervention phase and ideally optimize participation and adherence.

#### Health coaching

A growing body of research supports the effectiveness of the role a ‘health coach’ in chronic disease management [121, 123]. A health coach is a clinic-based health promoter trained to specifically stimulate and support initiation of and adherence to health behaviours (e.g., exercise, improved nutrition). Participants in the home-based arm of the intervention will work with a remote health coach, following a protocol adapted from Spring et al.’s successful trials in improving physical activity and weight loss [120, 121, 124, 125] as well as our prior experience in the phase II trial [101] and other trials by members of our group [126–129] (Additional file 3: Table S2). The health coach will meet with each home-based participant at study entry to help set goals and will make contact with participants every week to review uploaded exercise data in relation to goals. In addition to weekly contacts made via phone or electronic device, health coaches will meet face to face with participants once a month during the intervention period. This will support the development of alliance between participant and health coach and will include items that may not be possible in phone or electronic-based contact.

To enhance health coach-participant interactions, we will use specialized health promotion software for smartphones (an ‘app’) from NexJ Systems, Inc. (Toronto, Canada). Home-based study participants will receive smartphones, loaded with health monitoring and communication software and a wearable activity tracker (Fitbit) to use for the duration of the intervention. NexJ software effectively interfaces with Fitbit activity trackers to capture all of the features that the Fitbit tracks. Previous research demonstrates that Fitbit results are reliable, valid measurements of exercise adherence [130, 131]. These data will allow health coaches to better support participants, providing them with tools and suggestions for maintaining or increasing adherence based on Fitbit results.

#### Tapering

Tapering is designed to enable participants to achieve weekly exercise goals in an increasingly independent manner. Ultimately this will prepare participants to continue with regular exercise following the active intervention. To aid with this transition to independent exercise in the group arm, the number of weekly supervised exercise sessions offered to participants will be reduced from 3 to 2 in the fifth month and to 1 in the final month of the intervention. Participants will be asked to increase the number of independent exercise sessions to maintain a total of 4 to 5 sessions of exercise per week. In the home-based program, the frequency of contact with health coaches will be reduced from every week to every 2 weeks in months five and six and text message support will be reduced to 24-h turnaround time.

### Outcomes and measures

#### Co-primary outcomes

Our co-primary outcomes are fatigue, as measured by the Functional Assessment of Cancer Therapy–Fatigue (FACT-F) [132], and functional endurance, assessed with the 6-min walk test (6MWT). The FACT-F includes 13 items measuring cancer-related fatigue. It has excellent reliability and validity and published normative data [133]. Fatigue is a common symptom in men on ADT and fatigue in cancer patients is negatively correlated with many components of QOL [134–136]. It has been shown to improve consistently with exercise in prior trials in men on ADT [78, 79, 100]. The 6MWT is a commonly used, validated measure that assesses functional endurance [137, 138]. Physical fitness commonly declines with ADT use and improves with exercise [30, 68–70, 72, 73].

#### Secondary outcomes

##### Quality of life – General

General QOL will be assessed using the Functional Assessment of Cancer Therapy – General (FACT-G). It is a well-validated and widely used QOL measure that has been an outcome in major PC exercise trials [80, 139]. It can be completed in 8–10 min and has published normative data [140, 141].

##### Quality of life – Prostate-specific

Prostate-specific QOL will be assessed using the Functional Assessment of Cancer Therapy – Prostate (FACT-P) [142] which supplements the FACT-G with 12 prostate-specific items covering domains of urinary function, sexual function, pain, and related symptoms. It is also well-validated and has been used in multiple prior exercise trials [80, 142].

##### Physical fitness

Prior to the start of fitness testing, resting HR, BP, and oxygen saturation will be measured. Musculoskeletal fitness will be assessed using the 5-times sit-to-stand test, a common, simple, and validated measure of functional lower body performance and strength [143–145]. In addition to this, grip strength will be assessed as a measure of upper body strength [146] using a Jamar dynamometer and recording the highest of three readings for each hand. This measure is responsive to ADT use [12], and predicts long-term disability and mortality in middle-aged and older adults [147].

##### Body composition

Body fat percentage, fat-free mass, and fat mass will be measured using bioelectrical impedance analysis (BIA) with the Tanita TBF-300A device (Illinois, USA). Additional anthropometric measurements include waist circumference (WC), WC:hip ratio, and body mass index, following the standardized CSEP-PATH protocol [148]. In addition, BMD will be measured at lumbar spine, total hip, and femoral neck using dual x-ray absorptiometry (DXA).

##### Biological outcomes

Blood will be collected at three time points (baseline, 6 months, and 12 months) to analyze fasting blood glucose, lipid profile (total cholesterol, low density lipoprotein, high density lipoprotein, triglycerides), hemoglobin, glycosylated hemoglobin (HbA1c), total testosterone, as well as prostate-specific antigen (PSA) levels. These outcomes are being measured given the impact of ADT and exercise on various biological endpoints. More specifically, ADT leads to deleterious effects on blood glucose and cholesterol levels, whereas exercise may be associated with their improvement. Hemoglobin levels will be assessed since ADT leads to mild declines in hemoglobin that may affect fatigue, QOL, physical fitness, and adherence [70, 149, 150]. Glycosylated hemoglobin will be collected since regular exercise has been shown to reduce HbA1c levels whereas ADT is associated with worse insulin resistance and diabetic control [13, 52, 151]. Total testosterone levels will be collected to confirm that participants who are not actively on ADT during the intervention period are in an androgen-deprived state for the duration of the intervention. PSA is a standard safety measure, since there is theoretical evidence that exercise leads to an acute increase in testosterone that could aggravate PC [152]. In multiple prior exercise studies in men on ADT, PSA has been found to be unaffected following bouts of training or over the course of an intervention [30, 70, 73].

##### Costing and utility outcomes

To allow cost-effectiveness analysis, both the EQ-5D-5 L and a patient costs diary will be used. The EQ-5D-5 L is a well-validated, widely used, generic, off-the-shelf preference instrument that measures patient utilities across 5 dimensions of health (mobility, self-care, usual activities, pain/discomfort, and anxiety/depression) [153, 154]. It was originally known as the EuroQol, and has been widely used throughout the world in both clinical investigations and health policy determinations [155]. A total of 3125 unique combinations of scores across the 5 levels of each of 5 dimensions can be generated, each of which can be converted into a utility score based on population-weighted responses from either a United Kingdom-based population cohort or a United States-based cohort [154]. It can be completed in 5 min.

Resource utilization, out-of-pocket costs, and productivity costs will be estimated using a patient cost diary that we have used in prior PC studies, adapted for this study based on patient feedback and response analysis in the phase II trial. More specifically, the patient cost diary will capture: i) hospitalizations/medical events; ii) physician/other health professional services including hospitalizations and outpatient diagnostic tests; iii) drugs, including drug costs unrelated to ADT; iv) equipment costs; v) community services and home care; vi) productivity costs; vii) exercise-specific costs; and viii) out-of-pocket costs.

##### Adherence

Program adherence and predictors of adherence will also be assessed. We recognize there is no perfect, all-inclusive definition of adherence. We will therefore measure different elements of adherence in this trial. Our primary adherence measure is the proportion of individuals in each arm who achieve at least 150 min of MVPA at both the three-month and six-month time points based on objective measurements via accelerometry. A target of 150 min is recommended by both Cancer Care Ontario’s guideline for cancer survivors [117] and the revised Canadian Society for Exercise Physiology (CSEP) guideline for adults and older adults [156]. Recognizing limitations of accelerometry (e.g. technical malfunction, incomplete wearing, not capturing all activities) we will also capture self-reported MVPA with the Godin Leisure Time Exercise Questionnaire (GLTEQ) at each time point. The GLTEQ is a brief, self-administered validated questionnaire that records light, moderate, and vigorous exercise performed each week in 15-min increments [157, 158]. For those in the group supervised arm, we will capture weekly attendance at exercise sessions. For those in the home-based program, we will measure three elements of adherence/engagement: a) Fitbit step counts; b) frequency of health coach contacts; c) frequency of use of the smartphone app.

Study participants will be asked to wear Actigraph GT3X (Pensacola, FL) accelerometers daily from awakening to bedtime for 1 week at each assessment time point (including baseline). Accelerometers are reliable [159] and non-obtrusive, and capture all physical activity during waking hours, thereby providing information on total physical activity during the time period of observation [160]. Accelerometer data will be extracted in 60-s epochs and screened using standard methods for: (i) at least 4 days of valid data, including (ii) at least 10 h of wear time per day; (iii) continuous non-wear time (periods of time with zero movement counts per minute) of more than 1 h. Total time spent in MVPA (> 1952 movement counts per minute [161]) will be calculated for the week. Additionally, accelerometer data enables the capture of sedentary behaviour using a protocol of time spent in activities with < 100 counts per minute and time spent inclined (using the inclinometer setting).

**Fig. 2:**
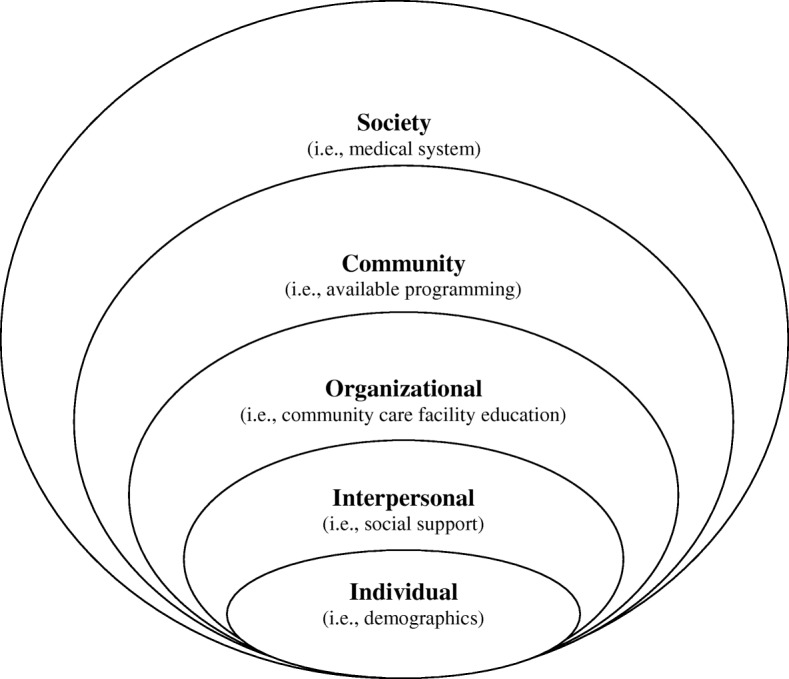
Social Ecological Framework for Understanding Exercise Determinants

Predictors of adherence were selected based on a social ecological framework (Fig. 2). Potential determinants of exercise adherence and the relationship between these determinants will be examined using validated measures at each level: exosystem, mesosystem, and microsystem. Exosystem will be examined via participant postal codes, which will be collected to map where participants live in order to obtain information about neighbourhood resources and environment. This will provide details about the walkability and access to recreation in participant neighbourhoods [162]. Mesosystem variables include assessment of autonomy support and social relatedness. We will use the Health Care Climate Questionnaire (HCCQ short form) [163], a 6-item self-report measure that assesses patients’ perceptions of the degree to which their health care ‘network’ is autonomy supportive. The modified HCCQ, which allows the assessment of provision of autonomy support from appropriate: (1) group members; (2) exercise instructors, and (3) health check ‘system’ [164] (i.e. 3 scales) will be used. Relatedness to Other Exercisers will be used as a social connectedness indicator of adherence using the Psychological Need Support and Frustration Scale – Relatedness Items [165]. This is an 8-item scale with good psychometric properties that assesses how individuals feel connected to other exercisers. The microsystem includes a measure of motivation, which will be assessed using the Behavioral Regulations in Exercise Questionnaire-2 (BREQ-2) [166, 167]. A modified version of this questionnaire that does not include the amotivation scale (4 items) will be used since participants who have consented to an exercise trial are likely not to be amotivated. The BREQ-2 is a 15-item inventory assessing extrinsic, introjected, identified, and intrinsic regulations. Planning, Attitudes, & Barriers will be assessed using a scale validated for cancer patients [168, 169].

##### Sedentary behaviour

An exploratory outcome will be change in sedentary behaviour, which is defined as **any waking activity characterized by an energy expenditure ≤ 1.5 metabolic equivalents** *and* **a sitting or reclining posture.** There are currently no data on men on ADT on levels of sedentary behaviour. These data will be obtained from accelerometry at the assessment time points.

### Safety

The CEP/RKin will review exercise precautions and safety during the intervention orientation. In addition, participants are encouraged to review the manual prior to commencing independent exercise sessions. All exercise personnel are trained in cardiopulmonary resuscitation (CPR) and automated external defibrillator (AED) use and have received safety training at each site. Possible adverse events during intervention and post-intervention phases will be captured using the National Cancer Institute Common Terminology Criteria for Adverse Events (CTCAE) v4.0. There is no independent data monitoring committee given the lack of anticipated safety concerns based on multiple prior trials of both interventions.

### Sample size calculation

A total of 200 participants (100 per arm) gives an estimated 81% power for analysis of the FACT-F and 6MWT for non-inferiority and will also provide accurate estimates of parameters related to secondary outcomes. Details are shown in Additional file 4: Methods. There are no planned interim analyses or early stopping rules.

### Statistical analysis

#### Co-primary outcomes

The primary analysis will use a Bayesian analysis of covariance (ANCOVA) model to regress the change in FACT-F and 6MWT values between baseline and 6 months against the baseline value, the treatment group variable and variables representing stratification by centre and length-of-ADT. We will treat the high-intensity group (group supervised; higher level of supervision and greater resources) as the reference.

**Fig. 3:**
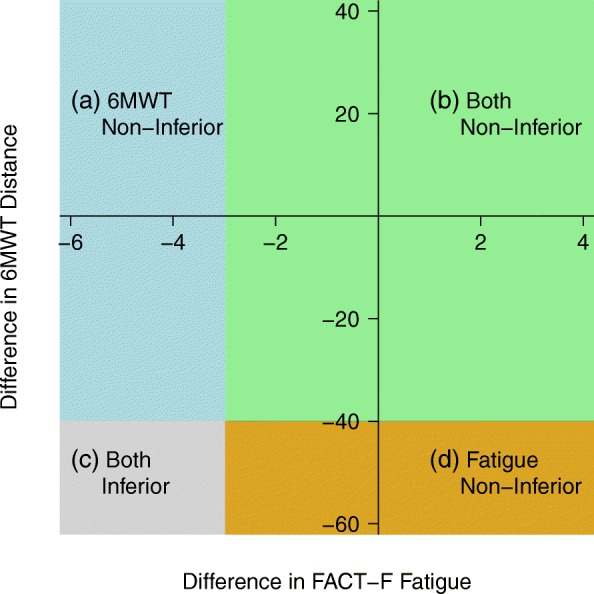
Illustration of Four Possible Scenarios in Non-Inferiority Testing for Proposed 2-Arm Trial. We will compare groups by means of ANCOVA using the baseline score as the covariate. After fitting a Bayesian model to each outcome, we will evaluate the probability in each of the 4 regions in the figure above. We can calculate the probability that: 1. Both the 6MWT and FACT-F fatigue scores are non-inferior in the home-based arm. This is the green region (**b**). 2. Both the 6MWT and FACT-F fatigue scores are inferior in the home-based arm. This is the grey region (**c**). 3. The 6MWT score is non-inferior in the home-based arm. This is the green region (**b**) plus the blue region (**a**). 4. The FACT-F Fatigue score is non-inferior in the home-based arm. This is the green region (**b**) plus the gold region (**d**). If either probability in (3) or (4) is above 97.5%, then we will declare non-inferiority of the home-based intervention.

For each primary outcome, we will compute the Bayesian posterior probability that the low intensity group is non-inferior to the high intensity group (i.e., that the mean difference between groups is larger than the non-inferiority margin -**δ**). If either of these probabilities is large enough (i.e. > 97.5%) then we will declare the home-based group to be non-inferior for that outcome. One advantage of a Bayesian approach is that it allows us to assign posterior probabilities to the 4 possible outcomes of the study (illustrated in Fig. 3): (1) Home-based inferior only for 6MWT; (2) Home-based inferior only for FACT-F; (3) Home-based inferior for both outcomes; and (4) both non-inferior. Another advantage is that it allows a more useful presentation of individual results than a simple confidence interval or *p*-value: for example, we will be able to say that the mean difference lies below –**δ** (i.e., home-based is inferior) with probability X%, that it lies between –**δ** and 0 (i.e., home-based is worse but non-inferior) with probability Y% and that it is above 0 (i.e., home-based is better) with probability Z%. Finally, the outputs of the Bayesian model can be used as probabilistic inputs for the economic analysis. The Bayesian analyses will use non-informative priors, allowing the results to be data-driven and make direct probability statements about non-inferiority.

Since this is a non-inferiority trial, the primary analysis will be per protocol, which in the presence of non-adherence to exercise is more conservative than intention-to-treat (i.e., less likely to conclude groups are similar when they are not). Two secondary analyses of the primary outcomes will be conducted. First, we will make the above between-group comparison of the changes in FACT-F and 6MWT scores from baseline to each follow-up time point. Secondly, we will perform an intention-to-treat analysis, including outcomes on subjects who were non-adherent to their exercise program. We will also conduct within-group analyses of change over time for the co-primary outcomes to understand the effects of each exercise modality on these outcomes and to facilitate comparisons with published studies.

#### Secondary outcomes

Analyses for each of our secondary outcomes (QOL, fitness, biological, and body composition) will use mixed effects models for data at all time points incorporating within-person correlation. Within-person correlation of residuals will be handled by an autoregressive (AR1) error structure and factors will also be added for the 2 stratification variables (centre, ADT duration). All of these secondary outcomes will be analyzed as continuous variables after examining distributions to ensure symmetrical (approximately normal) distributions. Skewed data will be transformed as appropriate.

#### Post-intervention period outcomes

Using similar mixed effects models, we will estimate changes over time and differences between exercise delivery groups in FACT-F and 6MWT during the 6-month post-intervention period, covering 3 assessment points (end of intervention, 3 months post-intervention, and 6 months post-intervention).

To give a quantitative overview of changes between and within groups over the entire 12 months, all study outcomes will be assessed using these same types of linear mixed effects models. By examination of estimated model parameters and their 95% confidence intervals, we can assess whether changes over time are similar between groups and whether differences between groups at any given time are clinically important.

#### Adherence

Several analyses will be used to assess adherence to the exercise programs and the relationship of adherence to both time and potential predictors. Adherence will be defined as a binary variable for the primary analysis, which uses accelerometry to measure MVPA. We will assess adherence over 4 distinct periods: 0–3 months, 3–6 months, 6–9 months and 9–12 months, so that each subject has up to 4 observation periods for adherence-based analyses. Our first analysis of adherence will simply be the calculation of percentages adhering in each group in each of the 4 time windows. Chi-squared or Fisher’s exact tests will compare adherence across groups within each of the time windows. All further analyses will be done on the sequence of 4 adherence measures on each subject using a generalized estimating equation (GEE) logistic regression model with an AR1 correlation structure.

Predictors of adherence will be examined at the exosystem, mesosystem, and microsystem levels using specific questionnaires as detailed earlier. These will be considered for inclusion in models examining each of the adherence variables separately. Mediation/moderation analyses will be explored based on the primary findings.

#### Cost-effectiveness

We will conduct a within-trial cost utility analysis using standard methods, focusing on interventions directly evaluated in the trial from both payer and societal perspectives. We will adopt two time horizons: the trial (12 months) and the lifetime of the trial cohort. Outcomes will be reported as quality-adjusted life years (QALYs) and cumulative costs, in undiscounted 2017 Canadian dollars.

#### Costs

We will estimate costs for all health care use in both arms, including:

i). hospitalizations/medical events;
ii). physician/other health professional services including outpatient diagnostic tests;
iii). drugs, including drug costs unrelated to ADT;
iv). equipment costs;
v). community services and home care;
vi). productivity costs;
vii). exercise-specific costs;
viii). out-of-pocket costs.

The intervention will be costed by estimating the value of time of those administering the intervention, facility-use costs, and device/ equipment costs, amortized over an appropriate period. Resource utilization, out-of-pocket costs, and productivity costs will be estimated using a patient cost diary. We will assess economically relevant health status using the EQ-5D-5 L at each time point [153, 154]. Valuation will be conducted using province-specific estimates when possible (e.g. schedule of physician benefits, cost per weighted case) and standard methods (e.g. provincial schedule of benefits, resource intensity weights and average cost per weighted case) [170–172]. Productivity losses will be valued both by the human capital (with adjustment for labour force participation) and friction cost methods [173].

#### Outcomes

Quality-adjusted life years (QALYs) will be estimated based on patients’ responses to the EQ-5D-5 L questionnaire [174] collected at each assessment. The EQ-5D-5 L provides a description of a patient’s health state, to which a utility score derived from a set of preference weights measured in a representative sample of the Canadian population can be applied. The EQ-5D-5 L weighted utility scores at each time-point will be used to estimate QALYs following standard procedures [175–177].

#### Analysis

Cumulative costs and QALYs for each trial arm will be estimated and compared in order to calculate the incremental cost utility ratio, and incremental Net Health Benefit. Censoring for both outcomes will be handled through inverse probability weighting [178, 179].

We will evaluate uncertainty and estimate confidence intervals around the estimates, using both deterministic and probabilistic methods. We will account for correlation between costs and health outcomes using appropriate bivariate methods. Cost-effectiveness acceptability curves (CEACs) will be used to graphically represent the probability that the intervention would be cost-effective for thresholds of $20,000, $50,000, and $100,000 per QALY gained (multiple thresholds for sensitivity analyses). Reporting will follow the Consolidated Health Economic Evaluation Reporting Standards (CHEERS) statement [180].

## Discussion

PC is the most common cancer in men. ADT is widely used to treat men with PC. It prolongs survival and slows disease progression but at the cost of numerous adverse effects. Exercise has been shown to mitigate and reverse many ADT-associated adverse effects, yet it is not widely available or implemented. To date, no study has examined which delivery model of exercise is most effective at producing these results or which are sustainable in the longer term, nor have studies examined the costs of these delivery approaches. These gaps in translational research must be addressed before exercise can be integrated into standard care for this population. The proposed multi-centre phase III trial aims to definitively answer these 3 questions (efficacy, long-term adherence, cost-effectiveness). Thus, this trial has tremendous empirical and practical relevance for men receiving ADT, and its findings should provide clear data on the value of implementing exercise programs for men on ADT. Additionally, our findings on psychological predictors of adherence will serve as the basis for future theory-based intervention studies designed to enhance long-term adherence to exercise. As such, the findings from the proposed study will serve as a foundation in the evidence-based clinical integration of exercise in this population.

As detailed elsewhere [113] (manuscript submitted), the current protocol is similar to our prior phase II trial in many ways, but with four key differences as informed by the results of the phase II trial [101]. First, the 1:1 supervised exercise arm has been eliminated. Second, all participants in the home-based arm will receive Fitbits to provide real-time, practical feedback on step counts as an easy to understand summary of physical activity performed. Fitbits were not provided in the phase II trial. Third, health coaches will meet once monthly in person with each participant in the home-based arm. In the prior trial, there was no in-person meeting. Fourth, to reduce participant burden in terms of outcome measures, and to incorporate emerging data on neighborhood walkability measures, our outcomes were streamlined with the Neighborhood Walkability Scale being substituted by postal code capture to facilite neighborhood walkability analysis, substitution of the PNSF for Relatedness to Others in Physical Activity Scale (ROPAS), and administration of the HCCQ at baseline only. These modifications should ensure the trial incorporates current evidence from published studies and our own phase II RCT, and positions it to provide invaluable data addressing the value of different exercise delivery models, determine the cost-effectiveness of exercise among men on ADT, as well as generate important insights into long-term adherence and predictors of adherence.

## Additional files

Additional file 1: CIHR ADT Ex RCT Protocol Paper SPIRIT Figure. Figure of study timelines (DOC 54 kb)

Additional file 2: **Table S1.** Study Participant Inclusion and Exclusion Criteria. Table describing study participant inclusion and exclusion criteria (DOCX 16 kb)

Additional file 3: **Table S2.** Elements of Health Coaching (adapted from Spring et al.). Table describing elements of health coaching. (DOCX 20 kb)

Additional file 4: CIHR ADT Ex RCT Protocol Paper Supplemental Methods. Sample size for 2-arm non-inferiority study with dual primary outcome. Methods used to determine trial sample size. (PDF 137 kb)

## Abbreviations

6MWT: Six-minute walk test
ADT: Androgen Deprivation Therapy
AED: Automated external defibrillator
BIA: Bioelectrical impedance analysis
BMD: Bone mineral density
BP: Blood Pressure
BREQ-2: Behavioral Regulations in Exercise Questionnaire – 2
CEP: Certified Exercise Physiologist
CHEERS: Consolidated Health Economic Evaluation Reporting Standards
CPR: Cardiopulmonary resuscitation
CSEP: Canadian Society for Exercise Physiology
CSEP-PATH: Canadian Society for Exercise Physiology – Physical Activity Training for Health
CTCAE: Common Terminology Criteria for Adverse Events
DXA: dual x-ray absorptiometry
EQ-5D: EuroQol 5 dimensions of health scale;
FACT-F: Functional Assessment of Cancer Therapy - Fatigue
FACT-G: Functional Assessment of Cancer Therapy - General
FACT-P: Functional Assessment of Cancer Therapy - Prostate
FITT: Frequency, Intensity, Time, Type
GEE: Generalized estimating equation
GLTEQ: Godin Leisure Time Exercise Questionnaire
HbA1c: Glycosylated hemoglobin
HCCQ: Health Care Climate Questionnaire
HR: Heart Rate
HRreserve: Heart rate reserve
MVPA: Moderate to Vigorous Physical Activity
PAB: Planning, Attitudes, & Behaviour questionnaire
PC: Prostate Cancer
PM: Princess Margaret Cancer Centre
PNSF: Psychological Need Support and Frustration Scale – Relatedness Items
PSA: Prostate-specific antigen
QALY: Quality-adjusted life years
QOL: Quality of Life
RC: Research Coordinator
RCT: Randomized Controlled Trial
RKin: Registered Kinesiologist
RPE: Rated perceived exertion
SRH: Scarborough and Rouge Hospital – Centenary site
SRHC: Southlake Regional Health Centre
TBCC: Tom Baker Cancer Centre
WC: Waist circumference
