# Supplementary material for: Protocol for a phase III RCT and economic analysis of two exercise delivery methods in men with PC on ADT
Source: BMC Cancer. 2018 Oct 23;18:1031. doi: 10.1186/s12885-018-4937-x (PMC6199786; doi:10.1186/s12885-018-4937-x)
Supplement: Supplementary file 1 — CIHR ADT Ex RCT Protocol Paper SPIRIT Figure. Figure of study timelines (DOC 54 kb) [file 12885_2018_4937_MOESM1_ESM.doc]

SPIRIT Figure - Schedule of enrolment, interventions, and assessments

|  | **STUDY PERIOD** | | | | | | |
| --- | --- | --- | --- | --- | --- | --- | --- |
|  | **Enrolment** | **Allocation** | **Post-allocation** | | | | **Close-out** |
| **TIMEPOINT**** | ***-t_1_*** | **0** | ***t_1_*** | ***t_2_*** | ***t_3_*** | ***t_4_*** | ***t_x_*** |
| **ENROLMENT:** |  |  |  |  |  |  |  |
| Eligibility screen | X |  |  |  |  |  |  |
| Informed consent | X |  |  |  |  |  |  |
| Allocation |  | X |  |  |  |  |  |
| **INTERVENTIONS:** |  |  |  |  |  |  |  |
| Group |  |  |  |  |  |  |  |
| Home |  |  |  |  |  |  |  |
| **ASSESSMENTS:** |  |  |  |  |  |  |  |
| Sociodemographic data |  |  | X |  |  |  |  |
| Quality of life questionnaires |  |  | X | X | X | X |  |
| Fatigue questionnaires |  |  | X | X | X | X |  |
| Adherence questionnaires |  |  | X |  | X | X |  |
| Body composition |  |  | X |  | X | X |  |
| Biological outcomes |  |  | X |  | X | X |  |
| 6 minute walk test |  |  | X | X | X | X |  |
| Timed chair stands |  |  | X | X | X | X |  |
| Grip strength |  |  | X | X | X | X |  |
| Accelerometry |  |  | X | X | X | X |  |
