# Supplementary material for: Protocol for a phase III RCT and economic analysis of two exercise delivery methods in men with PC on ADT
Source: BMC Cancer. 2018 Oct 23;18:1031. doi: 10.1186/s12885-018-4937-x (PMC6199786; doi:10.1186/s12885-018-4937-x)
Supplement: Supplementary file 2 — Table S1. Study Participant Inclusion and Exclusion Criteria. Table describing study participant inclusion and exclusion criteria (DOCX 16 kb) [file 12885_2018_4937_MOESM2_ESM.docx]

**Table S1: Study Participant Inclusion and Exclusion Criteria**

**Inclusion criteria**

1. histologically confirmed PC with an indication for ADT (high-risk/locally advanced disease, rising prostate-specific antigen (PSA) level after definitive therapy, or asymptomatic metastatic disease)
2. starting on or continuing ADT for a planned duration of at least 6 months or in an androgen-deprived (or castrate) state for the duration of the intervention.

*If individuals have been on ADT for <1 year, they are eligible to enter our trial if the period of coverage of ADT (i.e. date of final injection plus anticipated duration of the depot injection (LHRH agonists come in 1-month, 3-month, 4-month, and 6-month depots; most common is 3-month) is no more than 2 months* ***after*** *enrolment date. If individuals have been on ADT for >1 year, they are eligible if the period of coverage of ADT ended less than one month* ***before*** *enrolment date.*

*Since the recovery of testosterone after this time window is much less predictable, a serum total testosterone level of below 1.7 nmol/L within the last 3 months will be required to demonstrate ongoing castration and eligibility for trial enrolment.*

1. willing and able to provide informed consent
2. fluency in English
3. in proximity to one of the study centres

**Exclusion criteria**

1. engaging in regular moderate to vigorous physical activity at least 150 minutes per week (as assessed by the Godin Leisure Time Exercise Questionnaire (GLTEQ))
2. severe cardiorespiratory disease (including (a) severe coronary artery disease (Canadian Cardiovascular Society class III or greater); (b) uncontrolled hypertension (BP>160/95); (c) significant congestive heart failure (New York Heart Association class III or greater))
3. uncontrolled pain
4. neurological or musculoskeletal co-morbidity inhibiting exercise participation
5. history of poorly controlled major psychiatric illness
