# Supplementary material for: Protocol for a phase III RCT and economic analysis of two exercise delivery methods in men with PC on ADT
Source: BMC Cancer. 2018 Oct 23;18:1031. doi: 10.1186/s12885-018-4937-x (PMC6199786; doi:10.1186/s12885-018-4937-x)
Supplement: Supplementary file 3 — Table S2. Elements of Health Coaching (adapted from Spring et al.). Table describing elements of health coaching. (DOCX 20 kb) [file 12885_2018_4937_MOESM3_ESM.docx]

**Supplemental Table 2: Elements of Health Coaching (adapted from Spring et al.** **)**

We will follow a standardized protocol for health coaching adapted from Spring et al.’s successful trials in improving physical activity and weight loss^120, 121, 124, 125^. In two separate randomized controlled trials, Spring et al. included health coaches in one or more intervention arms to enhance behavioural modifications that led to increased physical activity and weight loss. Health coaches were paraprofessionals. The major elements were:

- 1. The health coach met with each study participant at study entry to introduce themselves and help the participant to set personal, achievable goals. Types of physical activities were guided by participant interest, skill, and level of physical conditioning.
  2. S/he made contact with participants every 2 weeks to review uploaded exercise data in relation to goals.
  3. Structured telephone contacts typically lasted 15 minutes.
  4. During telephone contacts, the health coach (a) provided support and encouragement; (b) discussed barriers to exercise; and (c) suggested additional exercises if the participant was ready to progress.
  5. Goals were progressed in a stepwise fashion, such that forward progression was mastery-based (i.e. patient proceeded to net goal only after the first goal was met).
  6. Additional exercises came from the modified Compendium of Physical Activities (modified by Ainsworth et al.^181^).
  7. Coaches also provided corrective feedback when implausible reports were uploaded, suggesting inaccurate (e.g. exercising for 12 hours per day) or untimely reporting (exercising for 3 hours starting at 3 am).
  8. Health coaches were also available 24x7 to provide telephone or text message support for technical problems with the smart phone, the app, or with uploading data.

The only modification we have made to the successful protocol by Spring et al. is to increase the intensity of contacts with health coaches to once weekly instead of once every two weeks, given the intensity of contact with trainers in the 2 supervised arms in our trial.
