# Supplementary material for: Protocol for a phase III RCT and economic analysis of two exercise delivery methods in men with PC on ADT
Source: BMC Cancer. 2018 Oct 23;18:1031. doi: 10.1186/s12885-018-4937-x (PMC6199786; doi:10.1186/s12885-018-4937-x)
Supplement: Supplementary file 4 — CIHR ADT Ex RCT Protocol Paper Supplemental Methods. Sample size for 2-arm non-inferiority study with dual primary outcome. Methods used to determine trial sample size. (PDF 137 kb) [file 12885_2018_4937_MOESM4_ESM.pdf]

Sample Size for 2-Arm Non-inferiority Study with Dual Primary Outcome

George Tomlinson

Tue May 24 2016

Methods

Assumptions for sample size calculations

We need to assume a non-inferiority boundary δ and the standard deviations of the fatigue score and 6MWT distance from the appropoperiate analyses for them.

1. δ(FACTF) = 3 ; SD(FACTF) = 10.5
2. δ(6MWT) = 40 ; SD(6MWT) = 110 ; Dropout = 10 %

I use the preliminary data from the Phase II study to estimate the correlation between baseline and follow-up measures to be 0.8 for the FACT-F fatigue and 0.5 for the 6MWT. The correlation between 6MWT and FACT-F fatigue at a given time is estimated as 0.2.

The non-inferiority boundary is 0.29 SD for fatigue and 0.36 SD for the 6MWT. To maintain a symmetry for the two outcomes, we will use a value of 0.4SD for each one.

We will compare groups by means of ANCOVA using the baseline score as the covariate. After fitting a Bayesian model to each outcome, we will evaluate the probability in each of the 4 regions in the figure below. We can calculate the probability that

1. Both the 6MWT and FACT-F fatigue scores are non-inferior in the homE-based arm. This is the green region (b).
2. Both the 6MWT and FACT-F fatigue scores are inferior in the homE-based arm. This is the grey region (d).
3. The 6MWT score is non-inferior in the home-based arm. This is the green region (a) plus the blue region (b).
4. The FACT-F Fatigue score is non-inferior in the home-based arm. This is the green region (a) plus the gold region (c).

If either probability in (3) or (4) is above 97.5%, then we will declare non-inferiority of the home-based intervention.

Under the assumption that the mean outcomes are the same in the two interventions, we can calculate the frequentist power as the probability that we will declare non-inferiority. Similarly, if we assume that the home-based intervention has an effect that lies on the non-inferiority boundary for both outcomes, we can calculate the frequentist typ I error as the probability that we will declare non-inferiority. The tables below show the results for these two extreme scenarios, as well as intermediate scenarios.

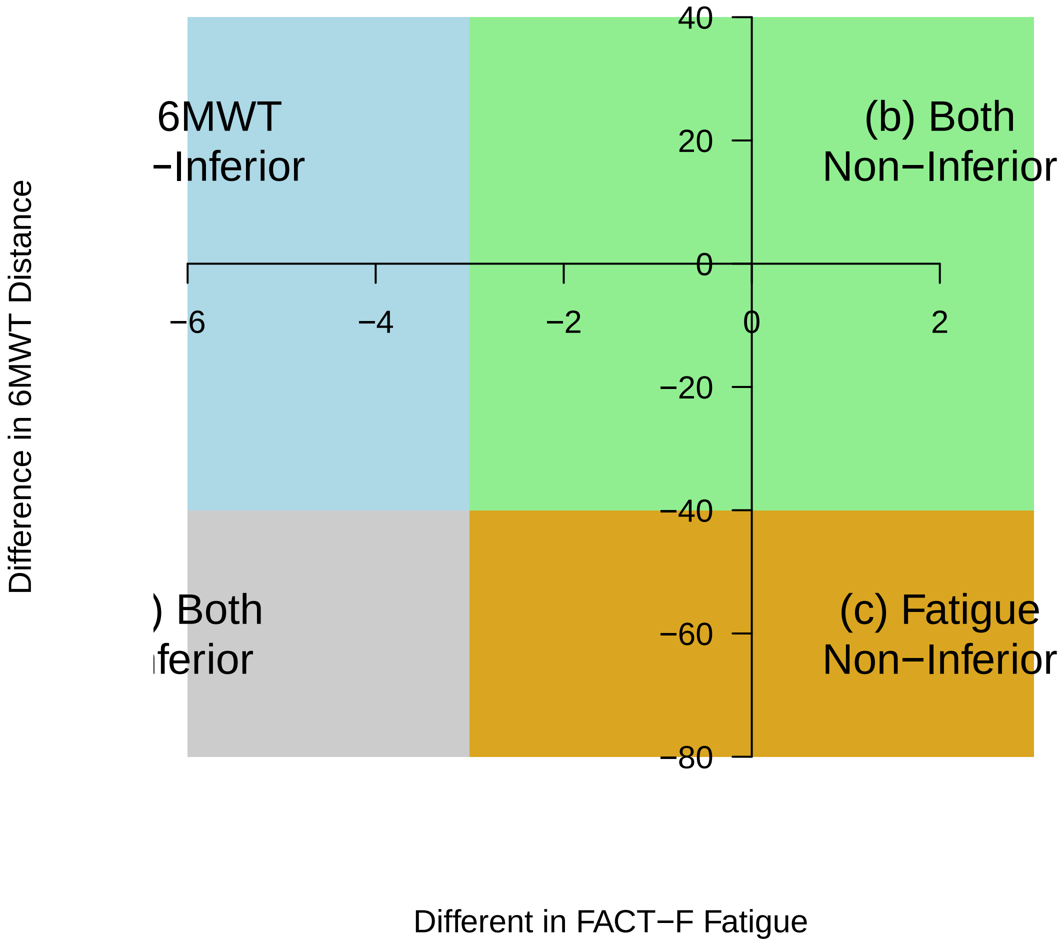

FATIGUE: Home = 0 worse and 6MWT Home = 0 worse.

Table 1: True diff in fatigue= 0 and true diff in 6MWT= 0

	BothNonInf	WalkNonInf	FatigueNonInf	OneNonInf
80	0.6946	0.7146	0.8058	0.9432
90	0.7498	0.7506	0.8574	0.9636
100	0.8048	0.7958	0.8882	0.9776
110	0.8532	0.8430	0.9174	0.9832

FATIGUE: Home = 0 worse and 6MWT Home = 40 worse.

Table 2: True diff in fatigue= 0 and true diff in 6MWT= -40

	BothNonInf	WalkNonInf	FatigueNonInf	OneNonInf
80	0.0434	0.0272	0.8080	0.8114
90	0.0440	0.0252	0.8486	0.8514
100	0.0456	0.0278	0.8908	0.8934
110	0.0420	0.0270	0.9120	0.9146

FATIGUE: Home = 3 worse and 6MWT Home = 0 worse.

Table 3: True diff in fatigue= -3 and true diff in 6MWT= 0

	BothNonInf	WalkNonInf	FatigueNonInf	OneNonInf
80	0.0350	0.7202	0.0250	0.7252
90	0.0410	0.7684	0.0264	0.7746
100	0.0406	0.7994	0.0256	0.8038
110	0.0378	0.8400	0.0238	0.8432

FATIGUE: Home = 3 worse and 6MWT Home = 40 worse.

Table 4: True diff in fatigue= -3 and true diff in 6MWT= -40

	BothNonInf	WalkNonInf	FatigueNonInf	OneNonInf
80	0.0010	0.0260	0.0240	0.0498
90	0.0012	0.0210	0.0278	0.0484
100	0.0010	0.0224	0.0280	0.0498
110	0.0014	0.0240	0.0286	0.0518
